# Oral chemolysis is an effective, non-invasive therapy for urinary stones suspected of uric acid content

**DOI:** 10.1007/s00240-020-01204-8

**Published:** 2020-08-07

**Authors:** Arman Tsaturyan, Elizaveta Bokova, Piet Bosshard, Olivier Bonny, Daniel G. Fuster, Beat Roth

**Affiliations:** 1 Department of Urology, Inselspital, University of Bern, Bern, Switzerland; 2 Department of General Medicine, First Moscow State Medical University after I.M. Sechenov, Moscow, Russia; 3 Department of Urology, Centre Hospitalier Universitaire Vaudois (CHUV), University of Lausanne, 1011 Lausanne, Switzerland; 4 Service of Nephrology, CHUV, University of Lausanne, Lausanne, Switzerland; 5 Department of Nephrology and Hypertension, Inselspital, University of Bern, Bern, Switzerland

**Keywords:** Nephrolithiasis, Oral chemolysis, Urine alkalization, Uric acid, Urolithiasis

## Abstract

Despite the possible benefit from avoiding stone surgery with all its possible complications, oral chemolysis is rarely performed in patients with urinary stones suspected of uric acid content. Among the reasons for its limited use is the sparse and low-quality data on its efficacy and the lack of reliable factors predicting its outcome. We thus performed a retrospective single-center cohort study of 216 patients (median patient age 63 years) with 272 renal (48%) and/or ureteral (52%) stones treated with oral chemolysis from 01/2010 to 12/2019. Patients with low urine pH (< 6), low stone density upon non-contrast enhanced computed tomography (NCCT), radiolucent urinary stones on plain radiography, and/or a history of uric acid urolithiasis were included. Potassium citrate and/or sodium/magnesium bicarbonate were used for alkalization (target urine pH 6.5–7.2). Median stone size was 9 mm, median stone density 430 Hounsfield Units. Patients with ureteral stones < 6 mm were excluded since stones this small are very likely to pass spontaneously. The stone-free status of each patient was evaluated after 3 months using NCCT. Oral chemolysis was effective with a complete and partial response rate of stones at 3 months of 61% and 14%, respectively; 25% of stones could not be dissolved. Lower stone density (OR = 0.997 [CI 0.994–0.999]; *p* = 0.008) and smaller stone size (OR = 0.959 [CI 0.924–0.995]; *p* = 0.025) significantly increased the success rate of oral chemolysis in multivariate logistic regression analysis. More precise stone diagnostics to exclude non-uric-acid stones could further improve outcome.

## Introduction

Urolithiasis is one of the most often diagnosed conditions in urology with a worldwide prevalence ranging from 5 to 10% [1]. Treatment of urinary stones varies from case to case depending on multiple factors including size, location, stone composition, and patient symptoms [2, 3]. Treatment can be conservative, non-invasive (shock wave lithotripsy [SWL]), minimally invasive (drainage with double J-stent or nephrostomy tube, endourological techniques) or, rarely, invasive (open or robotic). Most stones are composed of calcium oxalate, but about 10% consist of uric acid leading to an estimated prevalence rate of uric acid stones of up to > 0.75% [4]. The presence of uric acid in stones is associated with low density upon non-contrast enhanced computed tomography (NCCT), radiolucency on conventional radiology, and low urine pH [5]. Thus, historically conservative regimens that increase urine pH and thus can lead to dissolution of uric acid stones have been proposed for prevention and treatment of uric acid urolithiasis [6–9]. Nevertheless, and despite the possible benefit from avoiding stone surgery with all its possible complications, oral chemolysis is rarely performed—even if uric acid stone is highly suspected. Among the reasons for its limited use is the sparse and low-quality data on its efficacy. The aim of the present study is to evaluate the effectiveness of oral chemolysis (alkalization of urine) in the management of suspected uric acid stones and to identify possible factors that may improve patient selection.

## Methods

### Study population

**Fig. 1:**
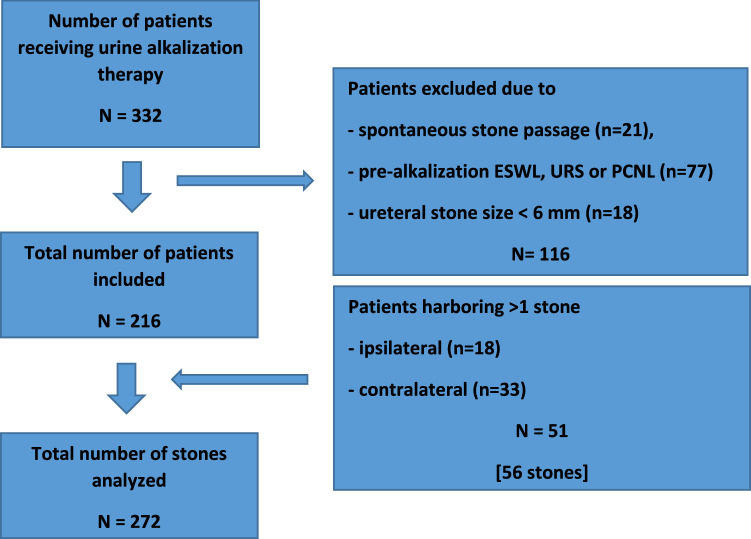
– Study flow chart

A total of 332 patients with renal and/or ureteral stones received oral chemolysis between January 2010 and December 2019 at our institution to dissolve suspected uric acid stones with low urine pH (< 6) [10], radiolucency on plain radiography, low density on NCCT (< 450 Hounsfield units [HU]) [11], recurrent uric acid stones, and/or presence of uric acid crystals on urinalysis. Patients receiving urine alkalization with preventive intent following complete interventional stone removal or after spontaneous stone passage were excluded from our study. In addition, ureteral stones of small size (< 6 mm) were excluded to avoid the bias that their high rate of spontaneous stone passage might misleadingly inflate the success rate of chemolysis [12]. As a result, data of 216 patients with a total of 272 stones were included in this study (Fig. 1).

### Intervention-urine alkalization

Oral chemolysis was performed using potassium citrate, sodium bicarbonate or magnesium bicarbonate. The selection of alkalization regimen was based on patient renal function, the tolerance of a given regimen**,** and whether they had a prior treatment of (recurrent) uric acid stones. Potassium citrate was indicated as a starting treatment regimen for patients with good renal function (eGFR > 0.7 ml/kg/min). An initial dose of 20 mEq three times daily was prescribed and adapted according to follow-up urine pH measurements. In patients with acute renal function impairment, intolerable pain and/or pyelonephritis due to an obstructing stone, treatment was only initiated after drainage (double J-stenting or nephrostomy) and return of serum creatinine levels and blood infection parameters to normal. If potassium citrate was not tolerated, it was replaced by sodium bicarbonate to complete the course of treatment. Patients with chronic renal insufficiency were given sodium bicarbonate as the initial treatment regimen. Magnesium bicarbonate was only prescribed for a single patient intolerant to both other regimens. For patients with hyperuricemia or a history of gout, xanthine oxidase inhibitor (allopurinol 100–300 mg/day) was added to the oral chemolysis regimen. Additionally, patients were advised to increase their fluid intake to at least 2.5 L/day. Metabolic work-up, however, was only performed after the stone treatment.

### Patient follow-up

All patients had NCCT [5] and pH measurement (dipstick) as baseline diagnostics and were followed-up according to our institutional protocol. Patients were meticulously instructed about regular urine pH measurement (three times daily using dipstick test of a spot urine sample) and adapting the drug dosage accordingly. The targeted urine pH was 6.5–7.2 [13]. A first follow-up visit with discussion of the patient’s urine pH diary and urine pH measurement was performed after 2 weeks. Patients receiving potassium citrate had an additional plasma potassium measurement. A second visit after another 2 weeks was requested of non-compliant patients only. Follow-up NCCT imaging was performed after the first 6 weeks of oral chemolysis. If required (partial dissolution only), another 6 weeks of oral chemolysis was administered followed by another NCCT imaging if deemed necessary. In case of symptoms due to the stone, intolerance of the medications, lack of stone dissolution, or at the patient's request, active stone removal was discussed with the patient at follow-up visits.

### Outcome measures and statistical analysis

Statistical analyses were performed using SPSS v. 21.0 (SPSS Inc., Chicago). Mean with standard deviation (SD) and median with range / interquartile range (IQR) were used to describe continuous and proportions of categorical variables, respectively. Stone size was defined as the largest diameter of the three dimensions on axial and coronal images of the pre-treatment CT scan. Stone density was measured using bone windows on the magnified axial NCCT of the stone at maximal diameter. If there were multiple ipsilateral kidney stones, stone size was defined as the sum of the maximal length of the stones. Ureteral stones and contralateral renal stones were considered as distinct stones and counted separately. Complete response and thus stone-free status was defined as the absence of any visible stone fragments on follow-up NCCT scan. Primary outcome was the stone-free status following urine alkalization therapy. Therefore, patients with no or partial response were analyzed together. When partial response was observed, the percentage of stone size reduction from initial stone size was calculated. Secondary outcome was to identify factors affecting the success of oral chemolysis using a univariate and multivariate logistic regression model. Independent variables were age, gender, BMI, eGFR, initial urine pH, drugs increasing uric acid excretion, stone location in the urinary system, stone size, stone density, pre-chemoytic intervention, chemolytic regimen, and duration of chemolysis. Only variables with a *p* value ≤ 0.2 on univariate analyses were included in the multivariate model. A two-sided *p* value < 0.05 was considered statistically significant.

## Results

The majority of patients were male (*n* = 162; 75.0%) with a median age of 63 years (Table 1). Median stone diameter was 9 mm (IQR 7–15), median stone density was 430 HU (IQR 380–500; Table 2). In more than half of patients (58.8%), a single course (6 weeks) of urine alkalization therapy was sufficient to reach the primary endpoint. In 11 patients (5%), the urine alkalization medication had to be changed or stopped due to side effects. Overall, a response to oral chemolysis was observed in 75% (204/272) of stones: complete in 61.0% (166 of 272) and partial in 14% (38/272). Dissolution > 50% was documented in 30% (11/38) of stones with partial response. Additional stone intervention [SWL, flexible ureterorenoscopy (URS) percutaneous nephrolithotomy (PCNL)] was required in only 22.1% (60/272) of stones (Table 2). Stone composition analysis by infrared spectroscopy was available in 30 patients undergoing additional intervention and revealed that 40% of these patients had stones without uric acid content (Table 3).

**Table 1:** Patient characteristics

Parameters	Total number of patients−(216)
Gender, *n* (%)	
Male	162 (75.0)
Female	54 (25.0)
Age (years), median (IQR)	63 (52—74)
BMI (kg/m^2^), median (IQR)	29.4 (25.9 – 33.8)
eGFR, median (IQR)	64.0 ( 48.0 – 81.0)
Gout, *n* (%)	
No	197 (91.2)
Yes	19 (8.8)
Initial urine pH, *n* (%)	
5.0	159 (73.6)
5.5	47 (21.8)
* ≥ *6.0	10 (4.7)
Alkalization specimen, *n* (%)	
Potassium citrate	202 (93.5)
Sodium bicarbonate	22 (10.2)
Magnesium bicarbonate	1 (0.5)
Duration of alkalization (weeks), *n* (%)	
6	127 (58.8)
12	89 (40.2)
Treatment interruption (intolerance), *n* (%)	
No	11 (5.1)
Yes	205 (94.9)
Xanthine oxidase inhibitors	
No	169 (78.2)
Yes	47 (21.8)

*IQR* interquartile range, *BMI* body mass index, *eGFR* estimated glomerular filtration rate

**Table 2:** Stone characteristics and treatment outcomes

Stone and treatment parameters	Total number of stones *n* = 272
Stone diameter (mm), median (IQR)	9.0 (7.0 – 15.0)
Stone location (kidney), *n* (%)	131 (48.2)
Renal pelvis	55 (20.2)
Upper calyx	34 (12.5)
Middle calyx	10 (3.7)
Lower calyx	10 (3.7)
Multiple locations	22 (8.1)
Stone Location (ureter), *n* (%)	141 (51.8)
Proximal (including uretero-pelvic junction)	93 (34.2)
Middle	21 (7.7)
Distal	27 (9.9)
Side of the stone burden, *n* (%)	
Left	170 (62.5)
Right	102 (37.5)
Intervention before alkalization, *n* (%)	
No intervention	89 (32.7)
Double J	175 (64.3)
Percutaneous nephrostomy	3 (1.1)
SWL	3 (1.1)
URS	1 (0.4)
PCNL	1 (0.4)
Stone density upon NCCT (HU), median (IQR)	430 (360—500)
Alkalization outcome at 3 months, *n* (%)	
No response	68 (25.0)
Partial response	38 (14.0)
Complete response	166 (61.0)
Reduction of size in 38 stones with partial response (% reduction), median (IQR)^a^	36.6 (20.0–61.8)
Stone intervention following oral chemolysis	
No further treatment	212 (77.9)
SWL	19 (7.0)
URS	33 (12.1)
PCNL	8 (2.9)

^a^Total number of stones with partial response = 38

*IQR *interquartile range, *HU *Hounsfield units, *NCCT *non-contrast enhanced computed tomography, *SWL *shock wave lithotripsy, *URS *ureteroscopy,* PCNL* percutaneous nephrolithotomy

**Table 3:** Stone analyses of 30 patients who required active stone treatment

Parameters	
Uric acid proportion, *n* (%)	
< 10	12 (40.0%)
10–49	2 (6.7%)
50–90	3 (10.0%)
> 90	13 (43.3%)

While univariate analysis identified stone diameter (OR = 0.945; CI 0.915–0.977; *p* = 0.001), stone density (OR = 0.997; CI 0.995–0.999; *p* = 0.007), stone location in the ureter (OR = 2.564; CI 1.553–4.233; *p* < 0.001), and pre-chemolytic intervention (OR = 1.781; CI 1.063–2.982; *p* = 0.028) as statistically significant parameters, only stone density (OR = 0.997; CI = 0.994–0.999; *p* = 0.008) and stone size (OR = 0.959; CI 0.924–0.995; *p* = 0.025) remained significantly associated with successful chemolysis in multivariate analysis (Table 4).

**Table 4:** Factors affecting chemolysis outcome on univariate and multivariate analyses

Variables	Univariate		Multivariate	
	OR (95% CI)	*p* value	OR (95% CI)	*p* value
Age	0.986 (0.969–1.004)	0.117	0.981 (0.961–1.001)	0.064
Gender				
Male	Ref			
Female	1.313 (0.748–2.304)	0.342		
Gout				
No	Ref			
Yes	1.303 (0.508 – 3.341)	0.582		
BMI	1.001 (0.963–1.04)	0.966		
Drugs increasing uric acid excretion				
No	Ref			
Yes	1.402 (0.751–2.62)	0.289		
Initial urine pH				
< 5.5	Ref			
≥ 5.5	0.962 (0.541–1.712)	0.895		
Stone diameter	0.945 (0.915–0.977)	**0.001**	0.959 (0.924–0.995)	**0.025**
Stone location				
Kidney	Ref			
Ureter	2.564 (1.553–4.233)	**< 0.001**	1.866 (0.984–3.539)	0.056
Stone HU	0.997 (0.995–0.999)	**0.007**	0.997 (0.994–0.999)	**0.008**
Pre-chemolytic intervention				
No	Ref			
Yes	1.781 (1.063–2.982	**0.028**	1.222 (0.639–2.337)	0.544
Chemolytic regimen				
Potassium citrate	Ref			
Sodium citrate	0.861 (0.532–1.391)	0.54		
Treatment courses (6 weeks)				
1	Ref			
2	0.827 (0.506–1.352)	0.45		

Bold indicates the result of statistical significance

*OR *odds ratio*, CI *confidence interval, *HU *Hounsfield units*, Ref *reference

## Discussion

We investigated the success rate of oral chemolysis in a large number of patients (*n* = 216) with renal and/or ureteral stones. The overall response rate (partial or total dissolution) was 75%; 61% of stones were completely, 14% partially dissolved after 3 months of oral chemolysis. Independent factors improving the outcome were lower stone density on NCCT and small stone size. Still, oral chemolysis was also effective in stones > 4 cm.

Uric acid stones rank as the second largest group of stones accounting for almost 10% of all urinary stones [4]. Up to 40% of all stones contain uric acid [5]. Active surgical treatments such as PCNL, URS and SWL are the mainstay modalities for patients with urinary stone disease regardless of stone composition. These techniques, however, are associated with a wide range of complications and contraindications. Conservative oral chemolysis represents a non-invasive alternative for treatment of stones with suspected uric acid content. In the present study, it had a high response rate of 75% and a low rate of 22.1% of secondary (active) stone intervention. Oral chemolysis was first described in 1933 by Violle [14]. Between then and 1980 several treatment regimens and dosing protocols were proposed [6, 7]. Thereafter—and following the advent of minimally invasive stone therapy—only a few studies have examined this particular treatment option [15]. Up to the end of 2019 most publications on this topic were of low quality and/or involved a limited number of patients. This paucity of data notwithstanding, both European and American guidelines recommend the use of urine alkalization regimens for treatment and prevention of uric acid stones [2, 16].

The reported success rates of oral dissolution therapy at 3 months range from 50 to 73% [17–21]. Some of the studies, however, included small-sized ureteral stones which might have passed spontaneously and thus account for a higher rate of “stone clearance” than our 61%. We therefore did not include ureteral stones < 6 mm in our study. Other studies had lower rates of complete dissolution. A prospective trial conducted by Elbaset et al. assessed the efficacy of oral dissolution therapy, SWL and a combination thereof [18]. For medium-sized renal stones from 1 to 2.5 cm, the authors reported stone-free rates of 16% at one month of oral chemolysis and 50% at 3 months. A similar stone-free rate (53.2%) at three month follow-up was reported by Elsawy et al. [20]. However, stones in their studies were larger compared to those in our study, supporting our finding that stone size significantly influences stone dissolution at pre-defined time points after start of oral dissolution therapy. Thus when oral chemolysis was administered for 6 months the stone-free rate increased to 83% [18]. For large stones, therefore, oral chemolysis should be prolonged to better achieve complete stone dissolution.

The main factors favoring the formation of uric acid stones are—besides genetic predisposition—acidic urine (pH < 6.0), hyperuricosuria, and low urine output [22]. Therefore, pharmacotherapy to increase the urine pH (urine alkalization) is recommended for successful dissolution of uric acid stones [5]. Currently, a urine pH of 6.5–7.2 is recommended for the treatment of uric acid stones [13, 23]. A further increase of urine pH should be avoided because it increases the risk of calcium phosphate stone formation [13, 24].

The efficacy of different alkaline solutions was examined in an in vitro experimental study by Heimbach et al. [25]. Potassium citrate is the most often used regimen for oral dissolution therapy**.** Its wide acceptance is based on evidence that potassium urate is more soluble in the urine than sodium urates [22]. While most of its side effects are of a mild gastrointestinal character (flatulence, abdominal pain, nausea, vomiting, diarrhea) and can be avoided with the simultaneous intake of sufficient liquid or with meals/snacks, a potentially dangerous side effect is hyperkalemia [4, 17]. Potassium citrate, therefore, should be avoided in patients with advanced renal insufficiency [24]. Sodium bicarbonate and sodium citrate represent alternative regimens [6].

Importantly, only 11 of our patients had to discontinue treatment due to intolerable side effects. Strict routine follow-up and wise consultation allowed us to monitor patients and detect those with hyperkalemia, to minimize side effects and ensure patients’ compliance with taking the prescribed medication.The knowledge of stone composition at diagnosis can greatly improve the effectiveness of oral chemolysis as it would exclude stones mistakenly thought to be of uric acid content. Novel technologies such as dual-energy CT can more accurately distinguish between uric acid and non-uric acid stones than conventional CT [26]. Because we used only conventional NCCT in our study, a high percentage of non-uric acid stone content (mainly calcium phosphate content) was present in 14 of 30 patients with available stone analysis and who had to undergo active stone removal. Since eight of these patients had a partial response to oral chemolysis (the other six had no response at all) before active treatment, we hypothesize that they had mixed-content stones and the observed partial dissolution was due to uric acid admixture. The other 16 patients with stone analysis after active stone removal had uric acid stone composition > 50%. More detailed analysis of these 16 patients showed that they were either partially non-compliant (*n *= 11) or developed side effects or intolerance that led them to stop the medication (*n* = 5). As a consequence, the urine of these 16 patients did not reach the target pH value of > 6.5 and oral dissolution therapy failed.

It has been shown that metabolic syndrome and obesity predispose to urinary stone formation. Particularly, uric acid stones are more prevalent in obese patients [1, 27]. Our cohort of patients had a high median BMI of 29.4 kg/m^2^ and 36 were even morbidly obese (BMI > 35.0 kg/m^2^). We found, however, no association between BMI and the success rate of oral chemolysis, an indication that oral chemolysis is also effective in obese patients. In contrast, for active treatment modalities such as SWL, URS and PCNL, higher BMI decreases stone-free rates and increases the incidence of postoperative complications [28, 29]. In light of this, oral dissolution therapy might be of special interest for this subgroup of patients.

To our knowledge, this study presents the largest cohort of patients with presumed uric acid stones treated with oral chemolysis alone and followed with NCCT imaging. Our study, however, has several limitations. The main limitation is its retrospective nature. Although our institution implements strict follow-up protocols to limit errors, potential bias cannot be fully eliminated. Another limitation is that ureteral stones that might have passed spontaneously were also included and might have positively influenced outcome. To minimize this potential bias, however, we excluded patients with small ureteral stones (< 6 mm). As a consequence, ureteral stone localization did not independently influence stone-free rate in multivariate analysis.

Another criticism might be that a high percentage of patients (64.3%) had undergone previous ureteral stenting (due to obstruction of the upper urinary tract) which is reported to significantly increase the success rate of oral chemolysis [30]. However, multivariate analysis did not reveal any influence of previous stenting on the stone-free rate.

## Conclusions

Oral chemolysis with urine alkalization is an effective and safe treatment modality for patients with ureter and kidney stones of suspected uric acid composition; complete response at 3 months was achieved in 61.0% of these stones. Lower stone density on CT and smaller stone size significantly improved the outcome of oral chemolysis. Most patients were spared active interventional stone therapy with all its potential complications; additional active stone treatment was only required in 22.1% of stones after unsuccessful complete oral chemolysis. More accurate stone composition diagnostics to exclude non-uric acid containing stones could further improve outcomes.

## Data Availability

At request under: beat.roth@chuv.ch.

## Code availability

SPSS v. 21.0 (SPSS Inc., Chicago).
